# Advanced Textile-Based Wearable Biosensors for Healthcare Monitoring

**DOI:** 10.3390/bios13100909

**Published:** 2023-09-27

**Authors:** Sheng Li, Huan Li, Yongcai Lu, Minhao Zhou, Sai Jiang, Xiaosong Du, Chang Guo

**Affiliations:** 1 School of Microelectronics and Control Engineering, Changzhou University, Changzhou 213164, China; sli@cczu.edu.cn (S.L.); hli9815@163.com (H.L.); yongcailu@foxmail.com (Y.L.); mhzhou2023@163.com (M.Z.); saijiang@cczu.edu.cn (S.J.); 2 CCZU-ARK Institute of Carbon Materials, Nanjing 210012, China; 3 School of Mechanical Engineering and Rail Transit, Changzhou University, Changzhou 213164, China

**Keywords:** textile, wearable biosensors, physiological indicators, health monitoring

## Abstract

With the innovation of wearable technology and the rapid development of biosensors, wearable biosensors based on flexible textile materials have become a hot topic. Such textile-based wearable biosensors promote the development of health monitoring, motion detection and medical management, and they have become an important support tool for human healthcare monitoring. Textile-based wearable biosensors not only non-invasively monitor various physiological indicators of the human body in real time, but they also provide accurate feedback of individual health information. This review examines the recent research progress of fabric-based wearable biosensors. Moreover, materials, detection principles and fabrication methods for textile-based wearable biosensors are introduced. In addition, the applications of biosensors in monitoring vital signs and detecting body fluids are also presented. Finally, we also discuss several challenges faced by textile-based wearable biosensors and the direction of future development.

## 1. Introduction

With the emergence of wearable technology and the exponential growth of biosensors, there has been a notable shift toward the advancement of a novel cohort of wearable biosensors built upon flexible textile materials [1,2]. This groundbreaking technology amalgamates the advantageous attributes of textiles and biosensors, opening up unprecedented avenues for medical monitoring, health management and the acquisition of biological data [3,4,5].

Conventional biosensors are commonly made from inflexible materials, which restricts their comfort and wearability [6,7]. Textiles, in contrast, possess qualities like softness, breathability and malleability [8]. Specifically, the softness of textiles permits the sensor to snugly conform to any part of the body for extended durations, maintaining continuous and comfortable touch with the skin, thereby facilitating the real-time monitoring of multiple vital signs [9,10]. Moreover, a range of functional materials, including inorganic nanomaterials, conductive polymers and electronic components, can be incorporated into the fabric structure using coating, printing or embedding techniques to create an integrated multi-parameter bio-detection system [11]. This textile-based biosensor platform maintains its sensing function while providing softness and breathability, resulting in an improved user experience and expanded applicability across different scenarios [12,13,14].

**Figure 1:**
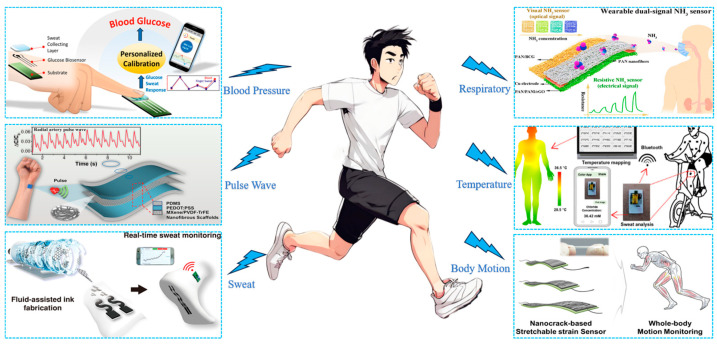
Recent advances in textile-based wearable biosensor for applications. Blood Pressure (Reprinted with permission from Ref. [40]. Copyright 2021, American Chemical Society (Washington, DC, USA)); Pulse ware (Reprinted with permission from Ref. [41]. Copyright 2020, American Chemical Society); Sweat (Reprinted with permission from Ref. [42]. Copyright 2022, American Chemical Society); Respiratory (Reprinted with permission from Ref. [43]. Copyright 2023, American Chemical Society); Temperature (Reprinted with permission from Ref. [44]. Copyright 2023, American Chemical Society); Body Motion (Reprinted with permission from Ref. [45]. Copyright 2017, American Chemical Society).

Textile-based wearable biosensors will drive the development of health monitoring, motion detection and medical management and become an essential support tool for individuals’ well-being, as illustrated in Figure 1. Textile-based wearable biosensors are capable of monitoring the body’s physiological indicators in real time, non-invasively. These indicators include respiratory rate [15,16,17], pulse [18,19,20], blood pressure [21,22,23], skin temperature [24,25,26] and humidity [27,28,29], which provide precise feedback information for an individual’s health. Real-time monitoring enables people to comprehend their physiological status, empowering them to take timely action. And they are widely used in the sports health field, providing personalized health advice by recording information such as exercise data [30,31,32], body posture [33,34] and energy consumption [31,35]. This helps users optimize their health habits and improve their quality of life. In addition, sensors hold a crucial position in the medical industry, serving as a helpful tool for disease detection and monitoring. They assist doctors with comprehensive and valuable data to enhance the management and treatment of diseases [36,37]. For elderly patients and those suffering from chronic illnesses, these sensors offer monitoring and alerting functionalities to improve their quality of life and respond to emergencies [38,39].

In this review, we begin by discussing recent advancements in textile-based wearable biosensors. Next, we outline the materials incorporated in biosensors. Then, the principles and methods for preparing wearable biosensors are introduced, followed by an overview of their applications in healthcare monitoring. Finally, we analyze the challenges facing textile-based wearable biosensors and discuss their future directions and application prospects.

## 2. Materials and Preparation Methods for Textile-Based Wearable Biosensors

### 2.1. Materials of Fabrication

Textile-based wearable biosensing technology offers new potential for human monitoring [46]. Wearable biosensors are fully flexible, unlike traditional silicon-based chip sensors, and can therefore easily adapt to human skin and movement, effectively collecting biological signals non-invasively for extended periods of time [47,48]. Wearable biosensors are typically composed of three basic layers: the substrate layer, the active layer and the interface layer. By utilizing various flexible materials, these three layers collaboratively carry out the entire process of acquiring bio-signals and outputting them into digital information [49,50,51].

Textiles containing fibers are natural, synthetic, functionalized and commonly used flexible substrates [52]. Natural fibers with inherent qualities and features are sourced from plant or animal sources. Common natural fibers like cotton and silk offer excellent moisture absorption, breathability and softness. Therefore, they are frequently utilized to create intimate apparel and sensor substrates, which can maintain comfortable skin contact for wearable biosensors. They must be combined with other conductive materials because of having no electrical conductivity [53,54]. On the other hand, artificially produced or chemically created fibers are known as synthetic fibers. The most popular synthetic fibers are nylon, polyester and acrylic. Through specific treatment, excellent qualities including conductivity, moisture wicking, strength and durability are obtained [55,56,57]. Additionally, functionalized fibers are given unique properties by incorporating unique elements into the fibers or applying surface modifications. For instance, conductive fibers are commonly utilized to transfer data and analyze biological signals. Luminescent fibers offer real-time visual feedback through incorporating luminous components into fibers [58,59,60,61].

The active layer can respond to external stimuli and convert them into measurable target signals through various sensing mechanisms [62,63]. Active layers are commonly made of various materials such as metallic nanomaterials, carbon nanomaterials, conductive polymers and ionic liquids [64,65]. Metallic nanomaterials, such as gold nanoparticles and silver nanoparticles, have excellent electrical conductivity and electrocatalytic properties. They can be used to prepare catalysts or electrodes for sensors that detect and change biomolecules. Surface modification and functionalization can also boost their interaction with biomolecules to enhance the performance of the sensors [66,67,68]. Carbon nanomaterials, such as carbon nanotubes and graphene, have excellent electrical conductivity, chemical stability and biocompatibility. By modifying their structure and surface functionalization, these materials are employed in electrodes or carriers of sensors and are adjusted for highly sensitive detection of certain biomolecules. Additionally, carbon nanoparticles are very biocompatible and have a large specific surface area [69,70,71]. Conductive polymers, such as poly(3,4-ethylenedioxythiophene) (PEDOT), polyaniline (PANI) and polypyrrole (PPy), are a class of polymeric materials with conductive properties. With good electrochemical activity and biocompatibility, they can be used as active layers or electrode materials for sensors. Conductive polymers are often used to regulate conductivity and biocompatibility by controlling the structure and doping impurities to achieve sensitive detection and analysis of specific biomolecules [72,73,74]. Ionic liquids are a class of liquids composed of ions with excellent ionic conductivity and chemical stability. Ionic liquids can function as electrolytes or carrier materials for sensors, increasing ion transport and promoting biomolecule–electrode interactions. They also possess a broad electrochemical window and low volatility, rendering them a potentially advantageous choice for biosensors [75,76,77].

The interface layer is responsible for connecting and transmitting signals, providing biocompatibility and protection. Additionally, it enhances the adhesion properties between the active layer and the flexible substrate, improving the stability of the sensor [78,79]. Common interface layer materials include polymeric materials, biocolloidal materials, bioactive nanomaterials, etc. Polymeric materials, such as polyvinyl alcohol (PVA), polyacrylic acid (PAA) and polydimethylsiloxane (PDMS), are frequently used for fabricating flexible, transparent sensor interface layers. They possess excellent biocompatibility and tunable physicochemical properties, which aid in reducing interfacial impedance between the sensors and biological tissues as well as in enhancing stability and biocompatibility [80,81,82]. Biocolloidal materials, such as gelatin and sodium alginate, have a porous structure and multifunctionality for improving the interaction between biomolecules and sensors. These materials are frequently employed as sensor interface layers to immobilize enzymes or biomolecules and to create three-dimensional microenvironments that are favorable for biological reactions [83,84,85]. Bioactive nanomaterials, including gold nanoparticles, magnetic nanoparticles and quantum dots, have large specific surface areas and unique biological properties that can enhance sensor sensitivity and selectivity. Additionally, these materials can immobilize biomolecules or serve as signal enhancers to enable efficient detection of biomolecules [86,87,88].

### 2.2. Methods for Fabrication

The effective integration of electronic materials into textiles is crucial for realizing the functionality of textile sensors [89,90]. At present, there are mainly three ways to give textiles electronic functions: (1) Attachment and embedding is the simplest preparation method, which is to attach microelectronic components or textile circuits to the surface of textiles or embed them into the interior to construct external e-textiles. (2) The coating or printing process integrates electronic materials into the surface of textile substrates and gives the textile electronic functions; it can be applied to the e-functionalization of ordinary fibers or fabrics. (3) Through the fiber preparation process, electronic materials can be directly processed into micro- and nano-structured e-fibers or fabrics. This method can prepare e-fabrics with more homogeneous overall electrical properties, and the internal structure design of e-fabrics can be more refined. These three preparation methods are discussed in detail below.

#### 2.2.1. Attachment and Embedding

To enable e-textiles to transmit data, process information and display/feedback signals, a straightforward approach is to affix firm microelectronic integrated circuits to the fabric substrate and construct electronic fibers that have logic operation functions within the fabric [91,92,93].

**Figure 2:**
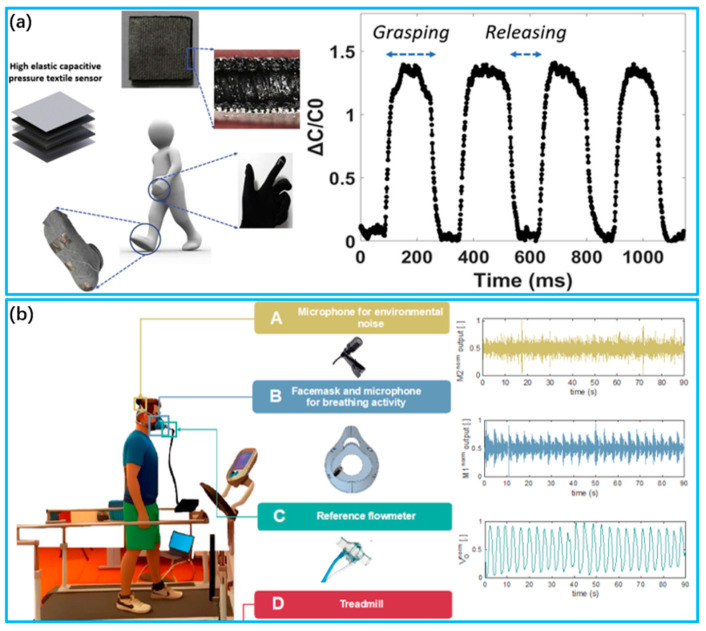
Preparation of wearable sensors using attachment and embedding methods. (**a**) Capacitive pressure sensor attached to a glove or sock for use in human movement monitoring (Reprinted with permission from Ref. [96]. Copyright 2020, Elsevier (Berlin/Heidelberg, Germany)); (**b**) 3D printed mask with microphone embedded for monitoring respiratory activity (Reprinted with permission from Ref. [97]. Copyright 2023, Multidisciplinary Digital Publishing Institute (Basel, Switzerland)).

Attachment technology is a method of securing a sensor assembly to a fabric surface. Common fixing methods include adhesives, hot melt glue and stitching. This approach is straightforward and convenient for small sensors and uncomplicated circuits [94,95]. However, e-textiles with attached electronic components are less comfortable to wear and cannot be washed. Kim et al. [96] presented a multipurpose capacitive pressure sensor with high sensitivity/elasticity and a very fast response time, as shown in Figure 2a. The pressure sensor was attached to a textile glove or sock for human movement monitoring.

Embedding technology entails incorporating the sensor component inside the fabric to safeguard the sensor via the fabric’s structure. This technology can offer superior durability and protective features while minimizing the impact on the fabric’s visual and tactile aspect [98,99]. Romano et al. [97] published a study in which they implanted a smart microphone sensor in a face mask to estimate the respiratory rate based on breath sounds during walking and running, as shown in Figure 2b. The signal-to-noise ratio of the breath signal was enhanced, and the impact of the external environment on motion detection was minimized.

#### 2.2.2. Coating and Printing

Constructing electrically conductive functional layers directly on the fabric surface is a facile and low-cost preparation method that retains the inherent softness of the fiber or fabric [100,101]. Coating and printing technologies play an important role in the fabrication of fabric sensors.

**Figure 3:**
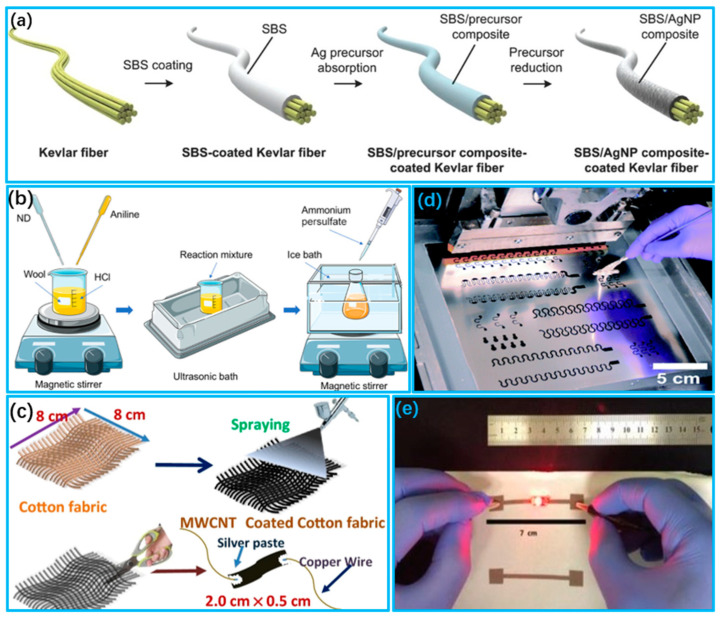
Preparation of wearable sensors using coating and printing methods. (**a**) Flow chart for the preparation of conductive Kevlar fibers via impregnation–reduction method (Reprinted with permission from Ref. [113]. Copyright 2015, Wiley (Hoboken, NJ, USA)); (**b**) Manufacturing technology of functional conductive wool fabrics with schematic representation of PANI and ND coatings on wool fabrics (Reprinted with permission from Ref. [115]. Copyright 2020, American Chemical Society); (**c**) Spraying MWCNT suspension on fabrics (Reprinted with permission from Ref. [118]. Copyright 2021, Springer); (**d**) Design templates for printing stretchable devices and screen printing processes (Reprinted with permission from Ref. [123]. Copyright 2016, Royal Society of Chemistry (Piccadilly, London)); (**e**) Inkjet printed e-textiles apply current to power LEDs (Reprinted with permission from Ref. [126]. Copyright 2019, Wiley).

The coating method is a common method for the fabrication of conductive textiles. The main conductive coating processes are impregnation coating [102,103,104], deposition coating [105,106,107] and spray coating [108,109,110]. Impregnation coating is accomplished by submerging the textile material in a conductive compound solution. This technique produces a conductive textile material that conducts electricity as a whole, demonstrating exceptional conductivity throughout [111,112]. Lee et al. [113] used a solution impregnation technique to create a uniform silver conductive film on a Kevlar fiber surface, as shown in Figure 3a. They utilized this film in an ultrasensitive and flexible pressure-sensing application. Deposition coating involves applying conductive materials onto textile surfaces to produce surface-conductive textiles. The resultant materials are only capable of conducting electricity across their surface [111,114]. Rehmen et al. [115] applied a coating of a composite material of polyaniline (PANI) with nanodiamond (ND) onto the surface of wool fabric via an in situ polymerization technique, as shown in Figure 3b. The resultant nanocomposite textile showed elevated sensitivity, durability and mechanical strength without diminishing the base properties of the material. Spray coating, on the other hand, is used to spray a layer of a conductive material onto the surface of the textile material by means of air spraying, electrostatic spraying, etc., so that the resulting conductive textile material is also surface conductive [116,117]. Maity et al. [118] prepared multi-walled carbon nanotube (MWCNT) modifiers using a spray coating method, in which the resistance of the MWCNT network on textiles could be altered by adjusting the MWCNT concentration and the quantity of spray coatings, as shown in Figure 3c.

The printing method enables the targeted placement of conductive elements onto fabric surfaces with improved controllability and flexibility compared to the coating method. Common printing methods include screen printing and inkjet printing [119,120,121]. Screen printing is a low-cost and straightforward preparation method that can easily be applied to any irregular fabric surface, providing greater design freedom and placement capability [122]. Jeerapan et al. [123] developed a scalable biofuel cell on a fabric surface through screen printing, as shown in Figure 3d. The original self-powered function can be maintained even when subjected to extreme mechanical deformation due to the synergistic effect between the functional ink and printed serpentine-like conductive lines. Inkjet printing has the potential to selectively functionalize textiles for electronic purposes. Inkjet printing theoretically reduces ink material usage and provides a higher spatial resolution [124,125]. Kim et al. [126] developed a conductive ink for inkjet printing on fabric that heats the fabric during printing, causing the silver particles in the ink to reduce and precipitate, as shown in Figure 3e. This enables the production of printed e-textiles with controlled conductivity and a high resolution, without the use of reactive particles.

#### 2.2.3. Spinning Technology

In spinning technology, pre-functional electronic materials are infused into the spinning precursor liquid, resulting in a flexible fiber with conductive capabilities through the spinning process. So far, the two most frequently utilized spinning methods are wet spinning and electrospinning [127,128,129].

**Figure 4:**
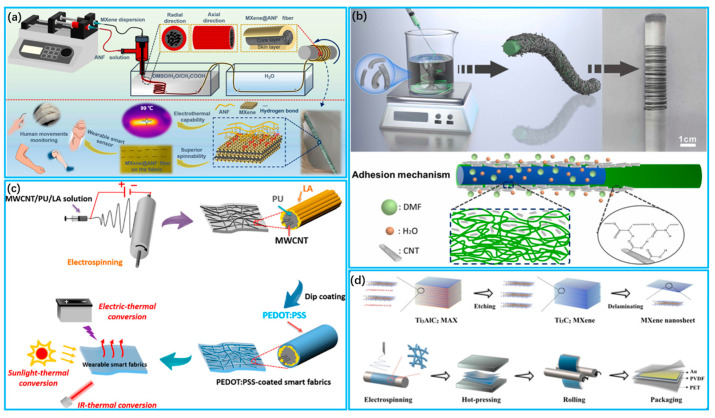
Preparation of wearable sensors using spinning technology. (**a**) Fabrication of MXene@ANF fiber via wet spinning process (Reprinted with permission from Ref. [132]. Copyright 2021, American Chemical Society); (**b**) Schematic diagram of CNT/TPU fiber wet spinning process and bonding mechanism (Reprinted with permission from Ref. [133]. Copyright 2023, Elsevier); (**c**) Fabrication of MWCNTs/LA/PU nanocomposite fibers via electrostatic spinning (Reprinted with permission from Ref. [136]. Copyright 2021, American Chemical Society); (**d**) Fabricated MXene/PVDF films were prepared via electrostatic spinning and other processes. (Reprinted with permission from Ref. [137]. Copyright 2023, Elsevier).

Wet spinning involves preconfiguring conductive materials into spinning solutions for spinning or preparing fibers with conductive precursors, followed by reduction to attain conductive fiber materials [130,131]. Wang et al. [132] used the simplified continuous wet spinning method to produce conductive MXene@ANF skin-core fibers, for which the high tensile and modulus ANF layer served as the backbone and skin layer, and the MXene material contributed to the excellent conductivity of the composite fiber, as shown in Figure 4a. Liu et al. [133] prepared highly stretchable composite fibers of carbon nanotubes/thermoplastic polyurethane (CNTs/TPU) via wet spinning, as shown in Figure 4b. The rotating solidification bath guaranteed full contact between the CNTs and TPUs. The resultant strain sensors showed an exceptional working strain range and fast response times.

Electrostatic spinning is based on the principle of electrostatic forces, and it is achieved by applying a high voltage to a polymer solution or molten polymer, resulting in the formation of elongated fibers under an electric field [134,135]. Niu et al. [136] prepared nanofibers via the electrostatic spinning of a multi-walled carbon nanotubes (MWCNTs)/lauric acid (LA)/thermoplastic polyurethane (PU) solution and adsorbed (3,4 ethylene dioxythiophene:styrenesulfonate) (PEDOT: PSS) on the obtained nanofibers, as shown in Figure 4c. This smart textile displays an adjustable temperature and phase change enthalpies and can respond to external stimuli such as voltage and light. Moreover, reversible storage and efficient conversion of energy are realized. Zhao et al. [137] utilized electrostatic spinning to prepare MXene/ PVDF hybrid films for highly sensitive flexible pressure sensors. The doping of MXene nanosheets facilitates the enhancement of the mechanical properties of PVDF, as shown in Figure 4d. With their excellent piezoelectric response and mechanical properties, MXene/PVDF thin-film sensors have outstanding voltage sensitivity.

## 3. Textile-Based Wearable Biosensors and Their Applications

Textile-based sensors combine the comfort, softness and wearability of textile materials with advanced sensing technology. They offer a convenient way for individuals to monitor their health in real time without needing extra devices or equipment. These sensors can be effortlessly integrated into day-to-day activities, sports training or medical management, playing a critical role [138,139,140].

### 3.1. Vital Signs Testing

#### 3.1.1. Tissue Pressure

In recent years, increasing attention has been paid to monitoring respiration, arterial pulse and blood pressure, as these fundamental physiological characteristics are directly connected to an individual’s well-being [141,142]. Real-time monitoring based on sensitive and efficient sensors allows for the timely acquisition of an individual’s physiological state and early detection of major diseases or emergencies. Tissue pressure data, including arterial pulse and blood pressure, are highly correlated with human physiological functions. The monitoring of tissue pressure holds potential for early diagnoses [143,144]. Therefore, pressure sensing is a critical function in wearable biosensors. Pressure sensors can be classified into four types based on their working mechanisms, including piezoresistive sensors, piezoelectric sensors, capacitive sensors and triboelectric sensors [145,146]. A comparison of the performance parameters of pressure sensors with different sensing mechanisms is indicated in Table 1.

**Figure 5:**
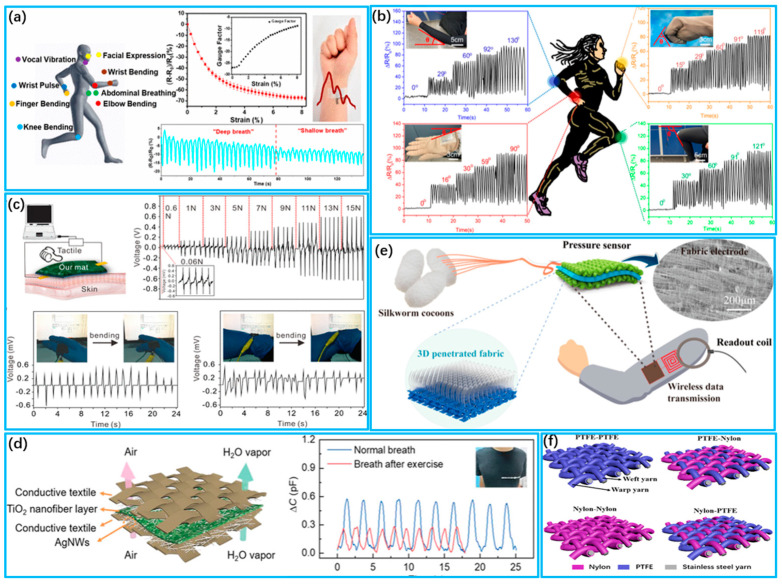
Detection of body tissue pressure by wearable pressure sensors. (**a**) Detecting various movements of the human body with wearable graphene fabric strain sensors (Reprinted with permission from Ref. [150]. Copyright 2018, American Chemical Society); (**b**) Monitoring flexion of body parts with AuNW/textile wearable sensors (Reprinted with permission from Ref. [152]. Copyright 2022, American Chemical Society); (**c**) Conductive MWCNT/PVDF−HFP nanofiber mats can be attached to human skin to directly detect tactile excitation and the output voltage of the nanofiber mats under different pressures (Reprinted with permission from Ref. [156]. Copyright 2019, American Chemical Society); (**d**) Schematic diagram of LC monitoring capacitive pressure sensor (Reprinted with permission from Ref. [139]. Copyright 2022, Wiley); (**e**) Schematic of breathable and wearable sensors and monitoring of respiration before and after exercise (Reprinted with permission from Ref. [160]. Copyright 2019, American Chemical Society); (**f**) Different structural designs of fabric pressure sensors and perception of body joint movements (Reprinted with permission from Ref. [163]. Copyright 2020, American Chemical Society).

Textile-based piezoresistive pressure sensors offer simple preparation, easy integration and convenient signal processing [147]. Researchers have made significant efforts to improve the performance of piezoresistive sensors by developing novel materials and designing various microstructures. Examples of such materials include graphene, carbon nanotubes, conductive nanowires and conductive polymers [148,149]. Yang et al. [150] fabricated a wearable graphene textile stress sensor with negative resistance change through the simple thermal reduction of GO, as shown in Figure 5a. Graphene oxide was utilized as a color additive to dye the polyester textile. This addition endowed the graphene textile strain sensor with excellent performance. Furthermore, the sensor exhibits remarkable properties, such as a wide strain range (up to 15%), high sensitivity and long-term stability. Park et al. [151] fabricated pressure sensors based on piezoresistive materials by inducing wrinkles on a shrink film. This process not only reduced stress but also enhanced pressure-sensing sensitivity by a factor of 12,800 while reducing the response time to less than 20 milliseconds. Zhao et al. [152] developed a highly sensitive textile-based piezoresistive sensor by layering gold-nanowire-embedded cotton textiles and silver ink screen-printed nylon textile electrodes, as shown in Figure 5b. The resultant sensing patch demonstrates superior performance, including a fast response time (load: 38 ms, recovery: 34 ms), a low detection limit (0.49 Pa) and high sensitivity (914.970 kPa^−1^, <100 Pa). It is capable of detecting respiration, pulse and blood pressure accurately during physical activity. Chen et al. [153] created strain sensors by immobilizing polypyrrole (PPy) onto knitted stretchable textiles made of polyester and spandex through low-temperature interfacial polymerization. The resulting sensors display excellent flexibility with a large dynamic range and high sensitivity, permitting the real-time monitoring of respiration and heart rate during exercise. This provides a uniform and dense coating method for conductive polymers on textiles that produces highly sensitive and stretchable sensors.

Textile-based piezoelectric pressure sensors rely on the property of piezoelectric materials to produce charge separation when subjected to an external pressure or force, which allows for the measurement and determination of the applied pressure to the sensor [154]. Piezoelectric sensors provide high sensitivity, a broad frequency response range, a rapid response time, a wide operational temperature range, corrosion resistance, durability and low power consumption for a diverse array of applications [155]. Li et al. [156] produced breathable and electrically conductive nanofiber mats that are highly flexible by utilizing electrostatic spinning techniques, as shown in Figure 5c. These textiles exhibited good electric conductivity, mechanical properties (up to 35.4 ± 7.3 MPa) and stability (≥10,000), enabling direct monitoring of human physiological signals. The successive inclusion of MWCNT into PVDF-HFP nanofibers showed a synergistic effect. A highly sensitive flexible pressure sensor based on piezoelectric MXene/PVDF hybrid films was proposed by Zhao et al. [137]. The doping of MXene nanosheets facilitates the enhancement of the mechanical properties of PVDF. The piezoelectric response and mechanical properties of the MXene/PVDF thin-film enable the sensor to demonstrate outstanding voltage sensitivity (S_v_ up to 0.0221 V/N)

Textile-based capacitive pressure sensors generally comprise parallel conductive electrodes and a dielectric layer that accumulates charge [157]. The sensing mechanism relies on changes in capacitance under an external force, which impacts the contact area, thickness and dielectric constant of the dielectric layer situated between the electrodes [158]. Moreover, as a wearable capacitive pressure-sensing device, the sensor should be flexible, comfortable, breathable, washable and durable [159]. Fu et al. [139] presented a piezocapacitive sensor with a flexible ceramic nanofiber network with high structural elasticity for real-time health monitoring, as shown in Figure 5d. It minimizes performance degradation in polymer-based sensors due to the viscoelastic behavior of polymers. It has high sensitivity (≈4.4 kPa^−1^), a low detection limit (<0.8 Pa) and a fast response time (<16 ms). Wu et al. [160] fabricated an all-textile wireless pressure sensor utilizing a 3D penetrating textile as a dielectric layer sandwiched between two highly conductive textile electrodes, as shown in Figure 5e. The sensor exhibited high sensitivity (0.283 KPa^−1^), high conductivity (0.33 Ω/sq), good stability (≥20,000) and excellent mechanical robustness. Zheng et al. [161] developed a capacitive sensor that is wearable and based on the capacitive coupling effect. This design improves the capacitive coupling effect and achieves high non-contact detection rates while having low sensitivity to tensile strain and pressure, compared to previous sensor designs.

**Table 1:** Comparison of performance parameters of pressure sensors with different sensing mechanisms.

Analyte	Materials	Sensor Mechanism	Fabrication Method	Response Time	Gauge Factor	Sensitivity	Stability	Ref.
Breathing, Pulse Wave	Graphene	Piezoresistive	Impregnation Coating	-	−26 (15%)	-	10,000 cycles	[150]
Pulse Wave	CNTs/PDMS	Piezoresistive	Shrinking Fabrication	<20 ms	-	278.5 kPa^–1^	-	[151]
Breathing, Pulse Wave	AuNW/textile	Piezoresistive	Impregnation Coating	<40 ms	-	914.970 kPa^–1^	30,000 cycles	[152]
Motion, Respiration	PPy/polyester/spandex	Piezoelectric	Spray Coating	<15 ms	−0.46 (0–71%)	-	500 times	[153]
Heartbeat, Motion	MWCNT/PVDF-HFP	Piezoelectric	Electrospinning	-	−0.7 (40%)	0.25 kPa^–1^	10,000 cycles	[156]
Breathing, Pulse Wave	MXene/PVDF	Piezoelectric	Electrospinning	3.1 ms	-	0.0480 V/N	-	[137]
Blood Pressure	TiO_2_ nanofibrous network	Capacitive	Electrospinning	<16 ms	−2.1 (60%)	4.4 kPa^–1^	50,000 cycles	[139]
Motion, Heartbeat	PVA/Ag NFs	Capacitive	Electrospinning	<10 ms	-	0.283 kPa^–1^	≥20,000 cycles	[160]
Pulse, Respiratory	LMs@PDMS	Capacitive	Stencil Printing	-	0.33 (0–50%)	0.0021 kPa^–1^	1000 cycles	[161]
Heartbeat, Respiratory	Conductive/nylon yarns	Triboelectric	Weaving	20 ms	-	7.84 mV Pa^−1^	>100,000 cycles	[162]
Pulse Wave, Motion	Nylon yarns/PTFE filament	Triboelectric	Twisting and Weaving	<15 ms	7.2 (30%)	1.33 V·kPa^–1^	4200 cycles	[163]

Textile-based triboelectric pressure sensors are capable of measuring the pressure distribution on body contact surfaces as well as sensing and monitoring pulses in a self-powered manner, without the requirement of an external power supply [164,165]. Fan et al. [162] reported a triboelectric textile sensor array (TSA) capable of capturing fine epidermal pressures. The TSA has various advantages, including high sensitivity (7.84 mV Pa^−1^), a fast response time (20 ms), high stability (>100,000 cycles) and machine washability (>40 washes). Additionally, it can monitor arterial pulse waves and respiratory signals simultaneously. The team also developed a smart health monitoring system that transmits the measured physiological parameters to a mobile application for CAD analysis and SAS assessment. For the complex and fine structure of friction nanogenerators, it is difficult to further improve the sensing ability of textiles within a limited contact area. To solve this problem, Lou et al. [163] developed a friction-based electric-sensing textile utilizing core-shell yarns. The positive and negative layers were made of nylon and polytetrafluoroethylene (PTFE) wires, respectively, and a spiral stainless steel wire acted as the inner electrode layer, as shown in Figure 5f. This innovative textile displays excellent sensing ability and mechanical stability. Moreover, it can be easily attached to the carotid artery to record arterial beat physiologic signals. This study provides a new approach for the development of highly wear-resistant textiles for large pressure sensors.

#### 3.1.2. Body Motion

The continuous monitoring of human joint motion in everyday environments is important for medical rehabilitation, healthcare and athletic performance [166]. Strain sensors play an important role in biomedical electronics to monitor a variety of body signals, including physical, chemical and biological signals [160,167]. Laminating strain sensors on human skin to detect bending or stretching is a frequent method in monitoring human activity [168]. Strain tactile sensors and pressure sensors have numerous benefits in this context, including a high tensile strength, a lightweight design, affordability, high sensitivity and a large range, and as a result, they are extensively employed [169,170,171]. A comparison of the performance parameters of different strain sensors for human motion is indicated in Table 2.

**Figure 6:**
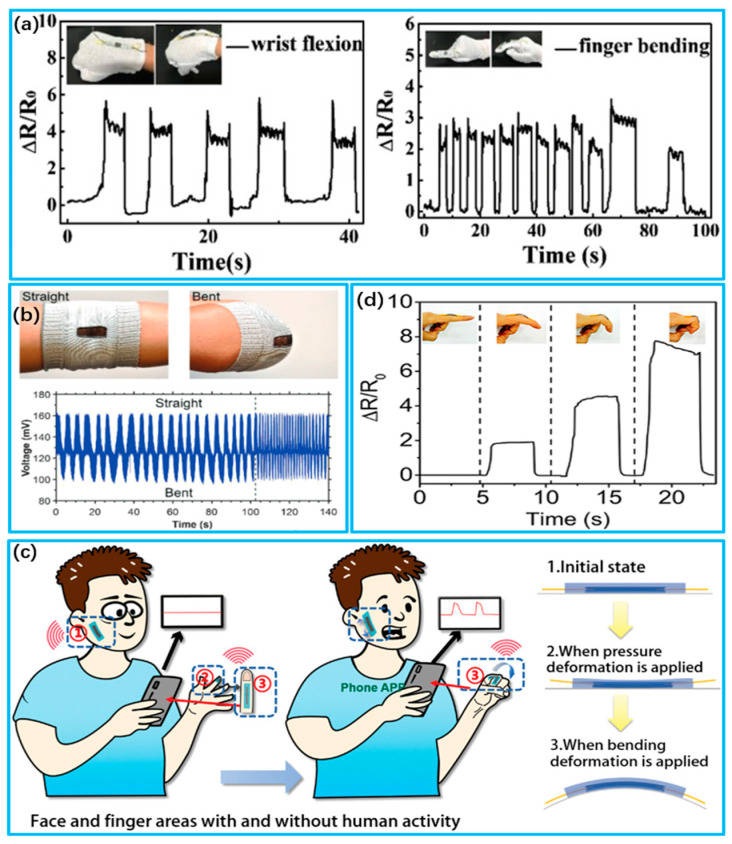
Wearable strain biosensors for human motion detection. (**a**) Detection of finger movement by RGOSF strain sensors (Reprinted with permission from Ref. [173]. Copyright 2019, Wiley); (**b**) Record of the strain-induced response of the MXene/PU sheath during bending and straightening (Reprinted with permission from Ref. [164]. Copyright 2020, Wiley); (**c**) Deformation states of flexible sensing devices under different human activity strains (Reprinted with permission from Ref. [175]. Copyright 2023, Wiley); (**d**) Relative resistance response of FSG strain sensors in detecting different degrees of finger bending (Reprinted with permission from Ref. [176]. Copyright 2016, American Chemical Society).

Evaluating strain sensor performance entails considering several crucial factors, including the sensing materials and device construction. These factors can significantly impact sensitivity, stretchability, response time and durability. Consequently, extensive efforts have been made to develop innovative manufacturing techniques and materials that can enhance strain sensor performance [164,172]. Materials including nanoparticles have been widely utilized in the field of stretchable strain sensing. Wang et al. [173] prepared graphene–silk textile strain sensors, as shown in Figure 6a. Graphene oxide deposited on silk textiles was converted into graphene to endow the textile sensor with electrical conductivity. Compared with other strain sensors, the sensor exhibits a linear and high resistive rate of change with increasing strain and can be used for the detection of human motion. Seyedin et al. [164] utilized the wet spinning technique to produce Ti3C2Tx MXene/PU composite fibers, as shown in Figure 6b. The resulting composite fibers display low conductivity at extremely low permeability thresholds, surpassing MXene-based polymer composites reported previously. They also exhibit high strain coefficients (~12,900) and large sensing strains (~152%). Yang et al. [174] constructed a self-powered laminated textile sensor using Schottky contacts. They sandwiched a polypyrrole (PPy)-coated textile between a nickel-coated textile and an aluminum–plastic film. The electrical output under strain is attributed to a single Schottky contact between PPy and Al.

**Table 2:** Comparison of performance parameters of different strain sensors for human motion.

Materials	Fabrication Method	Strain	Gauge Factor	Stability	Ref.
Graphene	Coating	10%	124	1000 cycles	[173]
MXene/PU	Wet spinning	50%	238	1000 cycles	[164]
		152%	12,900		
PPy/Al	Impregnation coating	25.3%	15	600 cycles	[174]
CNTs/PDMS	Drop coating	5%–30%	10	5000 cycles	[175]
		>30%	200		
RGO	Adhere	0%–60%	16.2	>5000 cycles	[176]
		>60%	150		

Recent studies have demonstrated that the stretchability and sensitivity of strain sensors can be improved through designing layered microstructures using a rational approach. This improvement addresses their reliability and accuracy in practical applications [177]. Du et al. [175] designed a stretchable multiple-stimulus responsive strain sensor based on a textile microstructure sensitive layer, as shown in Figure 6c. The sensitive layer of the sensor was designed as a textile microstructure. Carbon nanotubes were embedded in the microstructure region, and electrodes were added and encapsulated. The sensor exhibits excellent tensile, pressure and bending-sensing properties. Furthermore, it is proficient in recognizing human activities when placed on the skin. Liu et al. [176] reported a high-performance strain sensor with a fish-scale graphene sensing layer, as shown in Figure 6d. Changes in contact resistance are observed when the rGO slices in the initially overlapped area are stretched, enabling the detection of tensile and bending deformations with high sensitivity (strain coefficients ranging from 16.2 to 150), a wide sensing range (82% strain), a low detection limit (<0.1% strain) and excellent stability (>5000 cycles).

#### 3.1.3. Temperature and Humidity

Human body temperature and humidity need to be maintained within appropriate ranges to maintain normal body function and comfort [178]. Excessively high or low body temperatures as well as excessively high or low humidity may adversely affect human health; therefore, maintaining an appropriate body temperature and humidity is necessary for health and comfort, so much so that the real-time monitoring of body temperature and humidity is critical [179,180].

**Figure 7:**
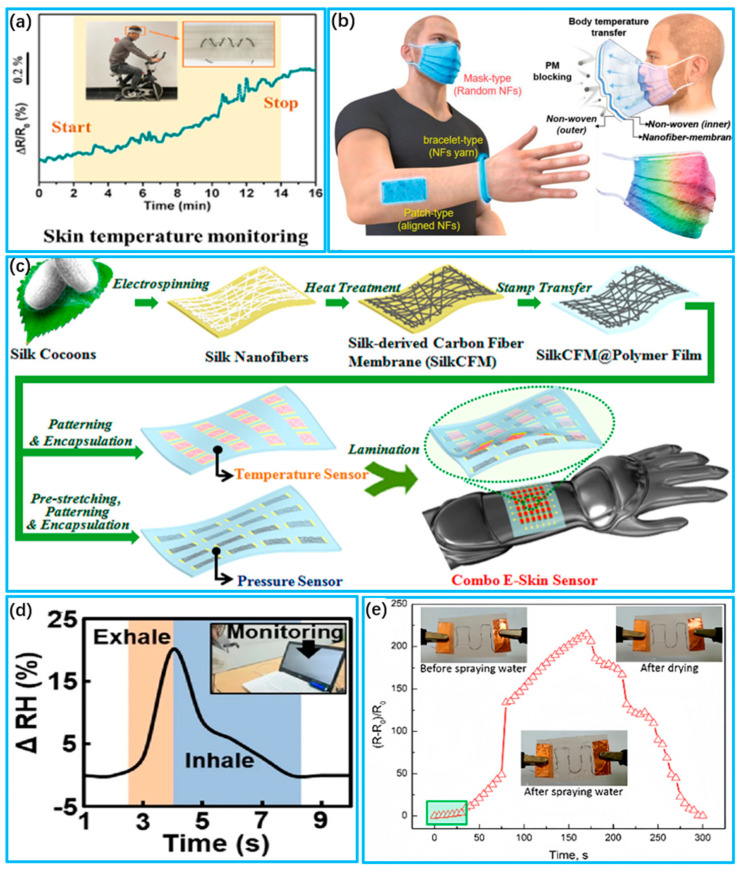
Wearable biosensors for human temperature and humidity monitoring. (**a**) Monitoring of body temperature during exercise with the PTCF temperature sensor (Reprinted with permission from Ref. [184]. Copyright 2022, American Chemical Society); (**b**) Body temperature monitoring using wearable thermochromic sensors for respiratory masks, bracelets and patches (Reprinted with permission from Ref. [185]. Copyright 2022, Wiley); (**c**) Fabrication process for silk-based combined temperature–pressure electronic skin sensors (Reprinted with permission from Ref. [186]. Copyright 2017, American Chemical Society); (**d**) Real-time respiratory measurements of deep breathing using a micro−controlled flexible humidity sensor (Reprinted with permission from Ref. [187]. Copyright 2022, Springer); (**e**) Resistance change of an SWCNT/PVA fiber sensor sewn to a highly hydrophobic textile before and after water spray (Reprinted with permission from Ref. [188]. Copyright 2017, American Chemical Society).

Temperature sensors are obtained based on materials with temperature-sensitive properties [181]. Conductive nanomaterials and resistive metals, such as gold nanoparticles and silver nanoparticles, are popular choices due to their high temperature tolerance and sensitive electrical properties [182,183]. Li et al. [184] fabricated a temperature sensor made of fibrous material. Thermoplastic polyurethane (TPU) fibers were used as the base material, and poly(3,4-ethylenedioxythiophene) (PEDOT) was grown in situ on their surface to produce a fibrous temperature sensor. The sensor possesses high sensitivity to temperature, a good temperature resolution (0.2 °C) and a high degree of linearity (0.998). The sensor can be used to monitor skin temperature during exercise, as shown in Figure 7a. Kim et al. [185] developed a wearable thermochromic sensor using electrostatic spinning technology. The nanofibers feature thermochromic properties and were created by adding an appropriate amount of thermochromic material to the spinning solution. These sensors can be applied to masks, bracelets and patches to monitor changes in body temperature in real time, as shown in Figure 7b. Wang et al. [186] reported a combined e-skin that measures temperature and pressure, using flexible and transparent silk nanofiber-derived carbon fiber membranes as active materials, as shown in Figure 7c. Temperature stimulation increases carrier hopping and induces tunneling conduction, leading to an increase in the conductance of the silk-based membrane. The silk-based temperature sensor demonstrates high sensitivity and a minimal response to external pressure stimulation.

Humidity affects humans’ experience with wearables. Too much humidity can cause symptoms such as increased body temperature, a rapid heartbeat, dizziness and fatigue, and too little can increase the probability of respiratory diseases [189,190]. Carbon nanomaterials, such as carbon nanotubes and graphene, have become the material of choice for humidity sensors due to their unique electrochemical properties and high specific surface area, and they have excellent mechanical properties and good flexibility [191,192]. Kim et al. [187] developed a low-resistance flexible humidity sensor based on CNTs. The sensor had a core–shell structure of CNT@CPM that was prepared with Chit and PAMAM, making it highly sensitive with an average response/recovery time of less than 20 s, as shown in Figure 7d. Zhou et al. [188] fabricated a humidity sensor using SWCNT/PVA filaments. The amount of SWCNT in the monofilaments can be adjusted to modify the conductive network of the CWCNT. Under humid conditions, the SWCNT/PVA filaments reach twice the diameter of the dry ones and exhibit high sensitivity at high relative humidity levels (RH), as shown in Figure 7e. Cho et al. [193] developed an rGO/SF humidity sensor with superior mechanical properties compared to rGO alone. The hybrid material exhibits up to a 232% higher elastic bending modulus and can be optimized by adjusting the reduction temperature. Li et al. [194] developed a capacitive humidity sensor utilizing a hybrid film comprising In_2_O_3_ and GO nanosheets. The integration of GO and In_2_O_3_ significantly improved the humidity-sensing capability. In_2_O_3_ has a cubic nanostructure, which is very favorable for the absorption and dispersion of water molecules by the In_2_O_3_/GO thin-film sensor.

### 3.2. Sweat Analysis

The human body contains multiple types of body fluids, including tissue fluid, saliva, sweat and so on. These fluids are closely related to potential diseases and maintain the normal physiological functions of the body. Unlike traditional medical methods (microneedle penetration, osmosis, pathological anatomy, etc.) for detecting body fluids, novel wearable fabric-based electronic devices have great potential for body fluid analysis, benefiting from their fast response and high efficiency [195,196]. Sweat is rich in physical information, more stable and easier to sample than tissue fluids such as tears, saliva and urine [197]. Today, fabric sweat sensors are mainly based on the principles of electrochemistry and colorimetry. Electrochemical biosensors convert the concentration of the substance into an electric current or voltage signals [198,199]. Colorimetric sensors determine the substance amount according to the color changing degree. Therefore, a wide range of biomarkers in sweat is detected, e.g., glucose, lactate, PH, cortisol, etc. Sweat sensors are useful for the monitoring of multiple biomarkers in sweat, as indicated in Table 3.

**Figure 8:**
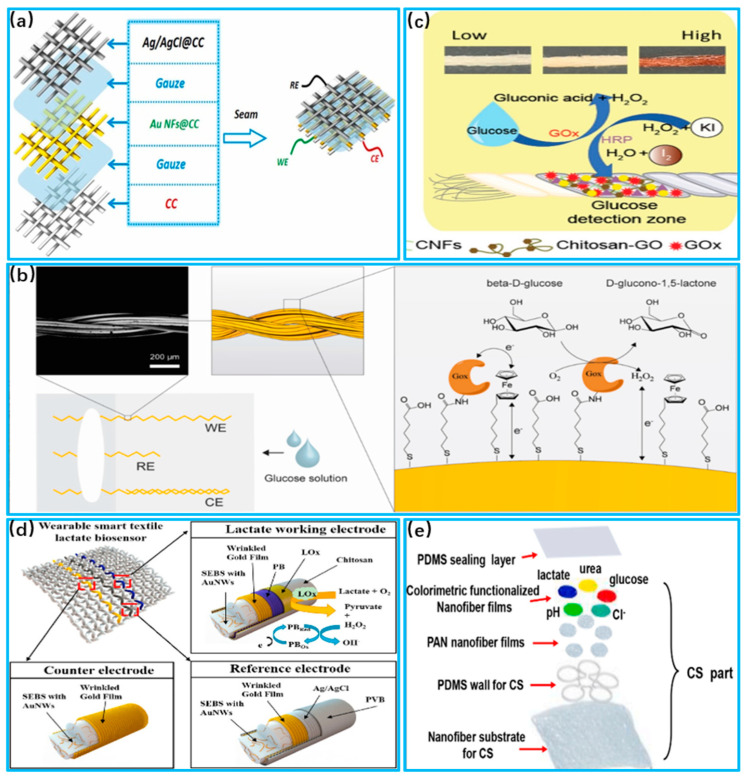
Application of sweat sensors in glucose and lactate detection. (**a**) Schematic of sensor based on coated carbon cloth (Reprinted with permission from Ref. [201], Copyright 2023, Elsevier); (**b**) Schematic representation of glucose assay chemistry performed with unstitched gold wires (Reprinted with permission from Ref. [202], Copyright 2021, Elsevier); (**c**) Systematic sensing mechanisms for glucose and urea detection (Reprinted with permission from Ref. [203], Copyright 2021, Elsevier); (**d**) Systematic sensing mechanisms for glucose and urea detection (Reprinted with permission from Ref. [204], Copyright 2023, Elsevier); (**e**) System fiber layer breakdown (Reprinted with permission from Ref. [205], Copyright 2023, Elsevier).

Glucose is one of the key molecules essential to the human body and is associated with energy and blood sugar levels. However, high concentrations can lead to diabetes, as well as complications such as kidney failure, blindness and stroke [200]. Zhao et al. [201] proposed a flexible enzyme-free sweat glucose sensor based on coated carbon cloth, as shown in Figure 8a. The sensitivity index of the sensor can reach 63.9μA/mM/cm^2^. Glucose reacts catalytically with the electrodes, generating a current between electrodes, which changes as the glucose concentration changes. Piper et al. [202] developed a wearable glucose detection device with stretchability by sewing conductive gold-plated threads into fabric, as shown in Figure 8b. Glucose reacts with oxidizing enzymes attached to the electrode to produce hydrogen peroxide. Hydrogen peroxide has electrochemical properties, and by comparing it to a reference electrode, the glucose content is determined according to the potential difference. In addition to electrochemical sensors, glucose can also be detected with colorimetric sensors. Promphet et al. [203] developed a wearable sensor based on cotton thread that can detect glucose concentrations in the range of 0.01−0.2 mM, as shown in Figure 8c. When the sensor’s detection zone comes in contact with sweat, oxidizing enzymes react with glucose to produce hydrogen peroxide. Hydrogen peroxide reacts with potassium iodide and results in a color change.

Lactic acid supplies energy to muscles to meet consumption demand, regulates the body’s acid–base balance and possesses a bactericidal effect in sweat [206]. Normal lactate levels in sweat are likely in the 5–25 mM range. However, high concentrations of lactic acid breaks the body’s acid–base balance and impairs the normal functions of cells [207]. Wang et al. [204] proposed an electrochemical sensor based on stretchable gold fibers for detecting sweat lactate levels, as shown in Figure 8d. The electrode is coated with lactate oxidase, which reacts with the lactate in sweat in the air to form pyruvate and hydrogen peroxide. Data on lactate levels are output by measuring the current of hydrogen peroxide during the redox process. Mei et al. [205] developed a lactate detection sensor based on electrospun nanofibers with an integrated colorimetric analysis, as shown in Figure 8e. The limit of detection (LOD) is 0.4 mm, and the limit of quantification (LOQ) is 1.3 mm. The nanofibers direct sweat into the sensor, and the lactic acid spreads out in the matrix to react with oxidative enzymes. Through the NFC chip inside the system, color images are analyzed with the help of a cell phone.

**Figure 9:**
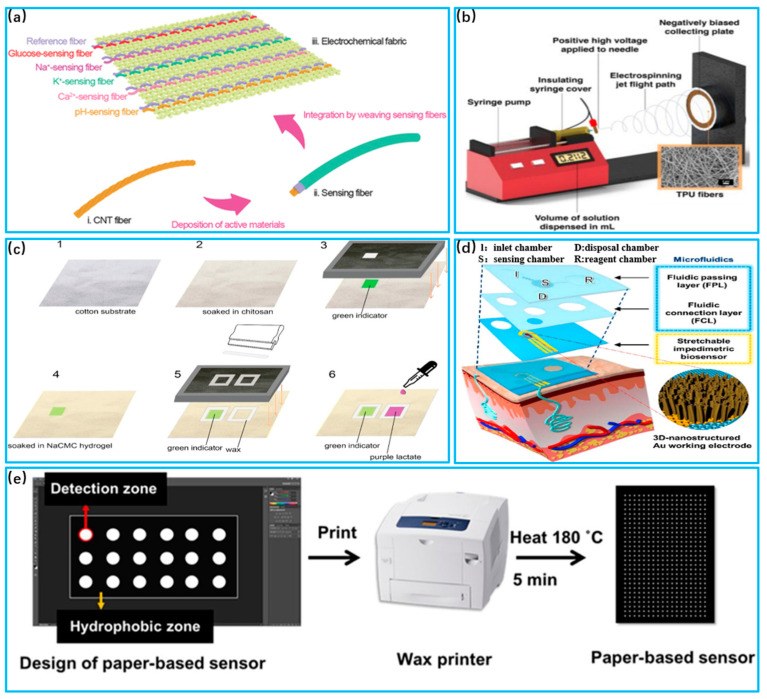
Sweat sensor for PH and cortisol applications. (**a**) Schematic of nanosensing fibers to fabricate electrochemical fabrics (Reprinted with permission from Ref. [210], Copyright 2018,Wiley); (**b**) Application of SERS activated AUTPU electrostatically spun wearable sweat PH sensor. (Reprinted with permission from Ref. [211], Copyright 2019, American Chemical Society); (**c**) Schematic manufacturing process of textile colorimetric sensors to detect sweat PH (Reprinted with permission from Ref. [212], Copyright 2019, Elsevier); (**d**) Schematic illustration of the fabrication of a paper-based sensor using a wax-printing method (Reprinted with permission from Ref. [213], Copyright 2020, Elsevier); (**e**) System fiber layer breakdown (Reprinted with permission from Ref. [205], Copyright 2019, Elsevier).

The PH value is one of the key factors in ensuring normal physiological activity in the human body. Only in a suitable range can proteins maintain their normal structure and function [208]. The PH level in sweat is usually between 4 and 7. Otherwise, a PH imbalance can lead to acid–base imbalance in the human body and can reduce the activity of other molecules [209]. Wang et al. [210] proposed an electrochemical sensor fabricated with sensing fiber units to monitor glucose concentrations within sweat, as shown in Figure 9a. The sensing fibers were constructed into glucose-sensing fibers by passing them through a Prussian blue membrane. Glucose oxidase was immobilized within the permeable membrane coated as the active layer of the sensor. Oxidase reacts with glucose, resulting in potential changes according to its concentration. Chung et al. [211] combined polyurethane electrostatic spinning materials and gold sputtering coatings to form a nanofiber flexible substrate, as shown in Figure 9c. The substrate was functionalized using responsive molecules. Sweat that flows through the substrate reacts with microelectrodes to measure the PH in sweat. Promphet et al. [212] proposed a wearable device to detect PH in sweat by preparing three different chemical layers on cotton fabric, as shown in Figure 9c. A colorimetric analysis was used to reveal different degrees of color as the concentration of PH increased, and they then compared it with a standard amount to derive the PH value.

Cortisol is a steroid hormone in the human body that regulates blood sugar and promotes the breakdown of proteins and fats. Excessive cortisol secretion leads to conditions such as high blood pressure and osteoporosis [214,215].Lee et al. [213] proposed an electrochemical sensor based on stretchable nanostructures to detect sweat cortisol, as shown in Figure 9d. Electrochemical impedance spectroscopy was utilized. The working electrode reads out the Faraday signal after the electrochemical reaction occurs, and the data of the cortisol content are obtained and combined with wireless data transmission. This sensor can stably detect cortisol levels from 1 pg/mL to 1 μg/mL, and the difference was about 14.7% compared to conventional testing. Apilux et al. [216] developed a paper-based sensor using a colorimetric analysis to detect sweat cortisol, shown in Figure 9e. A monoclonal antibody for cortisol was used as a signal indicator, and its contact with cortisol in sweat reveals different degrees of color. The degree of color represents different concentrations of cortisol, giving the user an intuitive feeling.

**Table 3:** Sweat sensors are useful for the monitoring of multiple biomarkers in sweat.

Detectives	Material	Response Time	Sensitivity	Detection Range	Limit of Detection	Ref.
Glucose	Au NFs	2~3 s	63.9 μA/mM/cm^2^	0.1~5 mM	-	[201]
Glucose	Conductive Gold Coated Threads	4~6 s	126 ± 14 nA/mM	0.01–100 mM	301 ± 2 nM	[202]
Glucose	Cellulose nanofiber	3~4 s	-	0.1~3 mM	0.1 mM	[203]
Lactic acid	Gold fibers	1~3 s	14.6 μA/mM cm^2^	0.1~5 mM	14.6 μA/Mm/cm^2^	[204]
Lactic acid	Nanofiber	1~2 s	70.3 nA Mm^−1^	-	0.38 mM	[205]
PH	Sensing Fiber	0.15~0.25 h	-	PH 4.0~7.0	PH 3.0	[210]
PH	Thermoplastic Polyurethane	1.5~2 h	0.14–0.33 PH	PH 5.5~7.0	PH 4.0	[211]
PH	Cotton Fabric	0.2~0.4 h	-	PH 1.0~14.0	PH 1.0	[212]
Cortisol	Nanometer	0.15~0.25 h	0.25 Ohm/ng mL^−1^	1 pg/mL~1 μg/mL	1 pg/mL	[213]
Cortisol	Paper Fibers	0.15~0.25 h	-	25–50 g/dL	21.5 g/dL	[216]

## 4. Conclusions and Outlook

In this review, we summarize recent advances in textile-based wearable biosensors, including materials, fabrication and devices. The characteristics of stretchability, comfort, breathability and biocompatibility from fabric materials enable biosensors to contact or fit well onto human skin, providing a more comfortable wearing experience. In the field of healthcare, textile-based wearable biosensors play an important role by enabling real-time, non-invasive monitoring of human physiological indicators, such as respiratory rate, pulse, blood pressure, skin temperature and humidity, which provide helpful suggestions for users. Therefore, more biochemical information through non-invasive monitoring is obtained, including metabolic levels, electrolyte balance and biomarkers of certain diseases from body fluids like sweat, tears and urine.

Despite the many potential applications of fabric-based wearable biosensors, there are still some technical defects and limitations, such as (1) the problem of target parameter selectivity and the signal interference of multimodal sensors. This problem concerns how to ensure that sensors make accurate measurements of specific physiological parameters without the influence of other parameters and the mutual interference of signals from multimodal sensors working simultaneously. This requires including the design of smarter signal processing algorithms and techniques to distinguish and eliminate interfering signals, as well as optimizing the selectivity of the sensors so that they can accurately measure the target parameters. (2) Stability and Durability: Textile biosensors are often subjected to frequent bending, stretching and washing environments, which may cause damage or failure of the sensor. Reliable sensor materials and construction must be designed to withstand daily use and washing. (3) Power and Communication: Textile biosensors typically require power and communication with external devices. However, integrating a battery and communication module may increase the weight and size of the sensor, which can affect user comfort. (4) Data Security and Privacy: Ensuring data security and privacy protection is an important challenge. The development of secure data transmission and storage schemes and the adoption of appropriate data encryption and authentication measures to protect the security of personal health data is an urgent issue. (5) User Acceptance and Adoption Rate: Factors such as user acceptance, comfort and ease of use play an important role in whether users are willing to wear and use sensors in the long term. Therefore, further efforts are required in designing user-friendly sensor interfaces and providing personalized user experiences, as well as continuous technological innovation and market promotion.

In summary, textile-based wearable biosensors are revolutionizing health monitoring, medical management and personal health management. We anticipate that these sensors will continue to innovate and evolve in the future and provide more accurate, convenient and personalized health support for human beings.

## Data Availability

In this review paper, no new data were created.
